# New insights into the intracellular distribution pattern of cationic amphiphilic drugs

**DOI:** 10.1038/srep44277

**Published:** 2017-03-10

**Authors:** Magdalena Vater, Leonhard Möckl, Vanessa Gormanns, Carsten Schultz Fademrecht, Anna M. Mallmann, Karolina Ziegart-Sadowska, Monika Zaba, Marie L. Frevert, Christoph Bräuchle, Florian Holsboer, Theo Rein, Ulrike Schmidt, Thomas Kirmeier

**Affiliations:** 1 Max Planck Institute of Psychiatry, Clinical Department, Munich, Germany; 2 Department Physikalische Chemie I, Ludwig-Maximilians-Universität München, Munich, Germany; 3 Max Planck Institute of Psychiatry, Department of Translational Research in Psychiatry, Munich, Germany; 4 Lead Discovery Center, Dortmund, Germany; 5 Nencki Institute of Experimental Biology, Warsaw, Poland; 6 HMNC GmbH, Munich, Germany

## Abstract

Cationic amphiphilic drugs (CADs) comprise a wide variety of different substance classes such as antidepressants, antipsychotics, and antiarrhythmics. It is well recognized that CADs accumulate in certain intracellular compartments leading to specific morphological changes of cells. So far, no adequate technique exists allowing for ultrastructural analysis of CAD in intact cells. Azidobupramine, a recently described multifunctional antidepressant analogue, allows for the first time to perform high-resolution studies of CADs on distribution pattern and morphological changes in intact cells. We showed here that the intracellular distribution pattern of azidobupramine strongly depends on drug concentration and exposure time. The mitochondrial compartment (mDsRed) and the late endo-lysosomal compartment (CD63-GFP) were the preferred localization sites at low to intermediate concentrations (i.e. 1 μM, 5 μM). In contrast, the autophagosomal compartment (LC3-GFP) can only be reached at high concentrations (10 μM) and long exposure times (72 hrs). At the morphological level, LC3-clustering became only prominent at high concentrations (10 μM), while changes in CD63 pattern already occurred at intermediate concentrations (5 μM). To our knowledge, this is the first study that establishes a link between intracellular CAD distribution pattern and morphological changes. Therewith, our results allow for gaining deeper understanding of intracellular effects of CADs.

The clinical efficacy of small molecules results from a complex interplay of various pharmacodynamic and pharmacokinetic processes and appears to be significantly influenced not only by regional (tissue specific) but also by (sub-)regional (intracellular) distribution patterns^1^. Distribution patterns of small molecules, in turn, are determined by their physicochemical properties^2^. While a plethora of information is available on how small molecules accumulate in tissues and in cells, significantly less is known about their exact subcellular localization^3^,^4^.

Cationic amphiphilic drugs (CADs) are well known for their potential to accumulate intracellularly in certain compartments. This drug family comprises a wide variety of substance classes such as antidepressants, neuroleptics, cardiac antiarrhythmics, and tranquilizers^5^. Common to all these molecules is their amphiphilic character that is brought about by hydrophobic head structures and hydrophilic side chains equipped with amines^6^. So far, the most common method for analysis of the intracellular accumulation pattern of CADs was the combination of radiolabeling of small molecules and differential centrifugation. It has been shown repeatedly that CADs, and in particular antidepressants, accumulate in compartments like synaptosomes, microsomes, mitochondria, and nuclei^7^,^8^,^9^,^10^. The major drawback of the use of radiolabeled compounds for intracellular CAD distribution assessment is the risk of accidental redistribution of the radioactive tracer into neighbor compartments. To overcome these obstacle, intrinsically fluorescent small molecules were tested regarding their suitability for monitoring changes in intracellular distribution pattern. Unfortunately, only a limited selection of intrinsically fluorescent CADs is available to date: amiodarone^11^,^12^,^13^,^14^,^15^,^16^, chloroquine^16^,^17^,^18^,^19^, clofazimine^20^, and quiancerine^14^,^21^,^22^,^23^ are the most frequently reported molecules. All four substances enrich in vesicular structures belonging to the endo-lysosome-secretory granule complex. However, low to moderate detection sensitivity and resolution made the quantification difficult. Further methodological alternatives for monitoring intracellular CAD distribution are Raman scattering microscopy and second ion mass spectrometry. In accordance with the findings obtained with intrinsically fluorescent molecules, Raman scattering microscopy revealed a predominantly intravesicular localization of CADs such as chloroquine and clofazimine^17^,^20^. Ion mass spectrometry, on the other hand, offers the potential of very high resolution and sensitivity but has so far not been employed in investigating intracellular distribution pattern of CADs. Although both of these methods represent promising methodological options, they come along with major technical challenges limiting their applicability^24^,^25^,^26^,^27^.

The aim of this study was to re-evaluate the intracellular distribution pattern of CADs using the recently synthetized and characterized CAD azidobupramine, a chemically modified antidepressant harnessing the power of modern medicinal chemistry to overcome above mentioned limitations^28^: azidobupramine carries an azide-group amenable for photoaffinity labelling (PAL) and an alkyne-group enabling chemical bonding of fluorescent tags by click-chemistry (CuAAC). These modifications allow for UV-induced immobilization of this CAD in living cells and subsequent for labeling with fluorophores without hampering subcellular structures. Here, we put a particular focus on the analysis of subcellular compartments which were previously reported to accumulate CADs. To achieve that, we employed cells stably expressing markers specifically targeting mitochondria, late endosomes and autophagosomes.

## Results

### Quantification of intracellular azidobupramine

**Figure 1:**
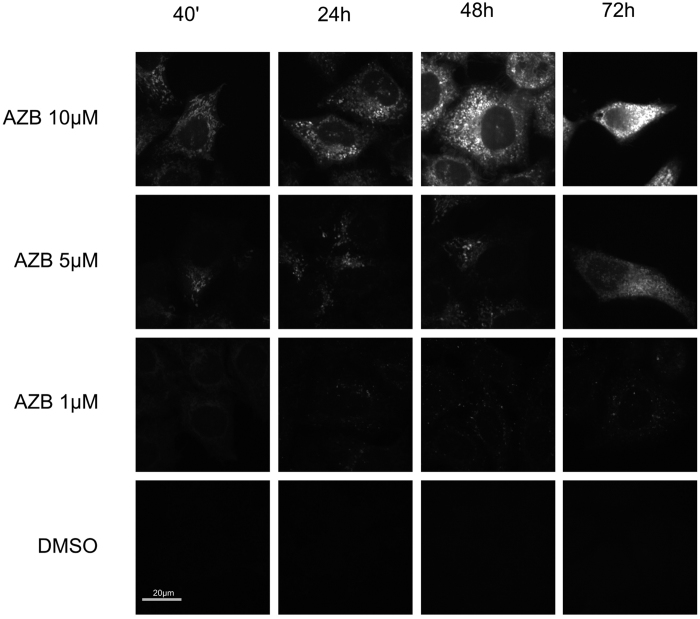
Intracellular accumulation of azidobupramine. For the detection of intracellular azidobupramine accumulation (white (false colored image)) Hela cells were exposed to different concentrations of azidobupramine (1 μM, 5 μM, and 10 μM) and different time periods (40 min, 24 hrs, 48 hrs, and 72 hrs) followed by an immobilization step (UV-light), and labeling of azidobupramine with fluorescent dyes by click-reaction; each picture represents a z-stag of one representative cell that was also the basis for the quantification; DMSO concentration was set to 0.1% in in all conditions.

**Figure 2:**
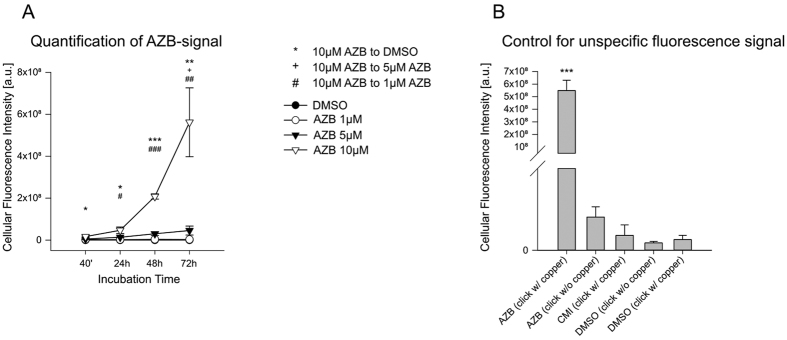
Quantification of intracellular azidobupramine. Quantification of the azidobupramine specific intracellular fluorescence signal at Hela cells treated as exemplified in Fig. 1: group analysis revealed higher cellular fluorescence under AZB 10 μM as compared to DMSO at all measurement times, as compared to AZB 1 μM after 24 hours, and as compared to AZB 5 μM after 48 hours; throughout the assessment, cellular fluorescence intensity became higher only under AZB 10 μM concentration (**A**). Control experiments for unspecific fluorescent signal: the fluorescence signal was measured after Hela cells were treated with 10 μM azidobupramine, clomipramine or DMSO over 72 hrs after UV-crosslinking and different click-reaction conditions with and without the copper-ion indispensable for click-reaction; One-way ANOVA revealed main effect of group (F(4, 10) = 44.26, p ≤ .001), with post-hoc analysis showing significant differences between AZB click with copper as compared to other variables at significance levels all at p ≤ .001 (**B**). *p < 0.05; **p < 0.01; ***p < 0.001; each data point represents the mean ( ± SEM) of three independent experiments comprising the integration and averaging of the fluorescent signal of 10 distinct cells; for more detailed statistical data please be referred to the supplementary material legend extension of Fig. 2.

First, we investigated the intracellular accumulation of azidobupramine (Figs 1 and 2A). HeLa cells were treated with azidobupramine at three different concentrations (1 μM, 5 μM, and 10 μM) over four different periods of time (40 min, 24 hrs, 48 hrs, and 72 hrs). The fluorescent signal was recorded after azidobupramine was immobilized on its target site by UV-crosslinking and the azidobupramine-target complexes were labelled with fluorescent dyes by click-chemistry (S-Fig. 1). Control cells were treated with equivalent volumes of the solvent DMSO. We observed that the fluorescence intensity increased both with time and increasing concentrations of azidobupramine indicating that the fluorescence signal measured was specific for azidobupramine (Fig. 2A). The Stokes Shift of all dyes used in the study were in the normal range of the Alexa dyes: excitation and emission could be separated clearly with our setup. Possible quenching effects at the highest dye-concentration could not be excluded ultimately but would render the observed effect even stronger than weakening it. Furthermore, we detected almost no fluorescence in cells treated with the control substances DMSO or clomipramine (Fig. 2B). The latter is a clinically active antidepressant which, in contrast to azidobupramine, is not chemically modified and hence does neither harbor an azido-group for UV-crosslinking nor an alkyne-group for click chemistry.

### Localization of intracellular azidobupramine signal

Next, we tested the intracellular distribution pattern of azidobupramine. We investigated the colocalization of azidobupramine with cellular organelle markers that have been described in context with a number of CADs and in particular with antidepressants^7^,^8^,^9^,^10^. For this, the intensity correlation quotient (ICQ) was calculated^29^. The ICQ can adopt values from −0.5 to + 0.5: −0.5 is indicative of complete anti-localization of the signal measured, + 0.5 indicative of complete co-localization, and 0.0 stands for random colocalization (S-Fig. 2)^29^. Three different cellular markers were employed: (a) the green fluorescent protein (GFP)-coupled tetraspanin CD63 (CD63-GFP) indicative of the late endosome-lysosomal compartment, (b) the GFP coupled microtubule-associated protein 1 A/1B-light chain 3 (LC3-GFP) indicative of the autophagosomal compartment, and (c) the red fluorescence protein expressed in the mitochondria (mDsRed) indicative of the mitochondrial compartment.

**Figure 3:**
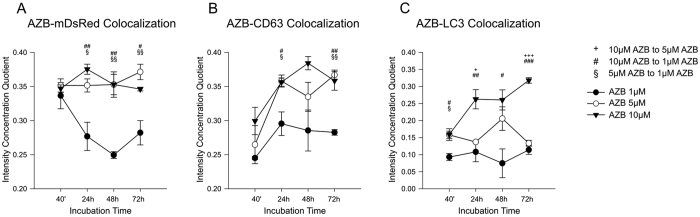
Analysis of compartment specific distribution pattern of azidobupramine related to concentrations used. *Colocalization of azidobupramine with the mitochondrial compartment was analyzed with two-way ANOVA with repeated measures*: after 24 hours, the intensity correlation quotient was lower under AZB 1 μM in comparison to AZB 5 μM as well as to AZB 10 μM; throughout the assessment, time-related changes were observed only under AZB 1 μM (**A**). *Colocalization of azidobupramine with the endo-lysosomal compartment was analyzed with two-way ANOVA with repeated measures*: group analyses revealed at both measurement times 24 hours and 72 hours significant differences between AZB1 μM as compared with AZB5 μM as well as with AZB10 μM (**B**). *Colocalization of azidobupramine with the autophagosomal compartment was analyzed with two-way ANOVA with repeated measures*: group differences were observed between AZB 1 μM and AZB 10 μM at all measurement times; moreover, intensity correlation quotient for AZB 1 μM was lower than AZB 5 μM at 40 minutes and for AZB 5 μM was lower than AZB 10 μM at both 24 hours and 72 hours (**C**). *p < 0.05; **p < 0.01; ***p < 0.001; each data point represents the mean ( ± SEM) of three independent experiments averaging the signal of 10 distinct cells; for more detailed statistical data please be referred to the supplementary material legend extension of Fig. 3.

**Figure 4:**
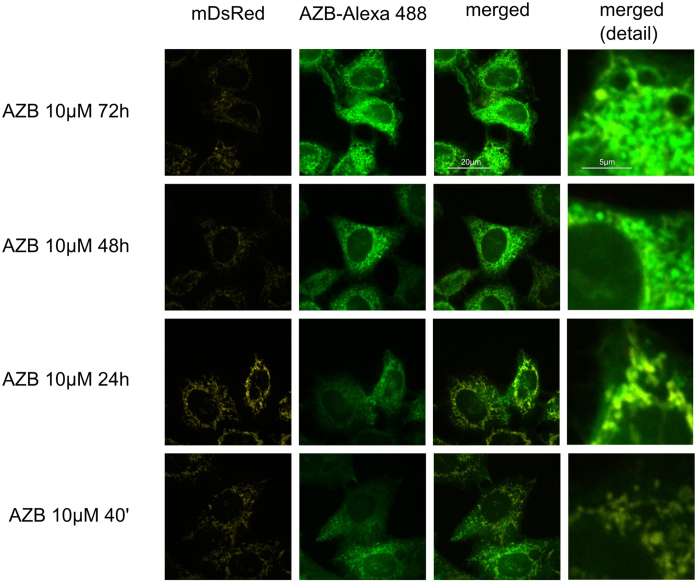
Colocalization of azidobupramine with the mitochondrial compartment. Hela cells stably expressing mDsRed a marker protein (yellow) for the mitochondrial compartment were exposed to azidobupramine at 10 μM (Alexa Fluor 488, green) for varying time periods (40 min, 24 hrs, 48 hrs, and 72 hrs) followed by UV-light crosslinking and click-chemistry; the colocalization of azidobupramine with mDsRed was most prominently detectable after 40 minutes and 24 hours and less obvious at 48 hours and 72 hours; DMSO concentration was set to 0.1% in in all conditions.

**Figure 5:**
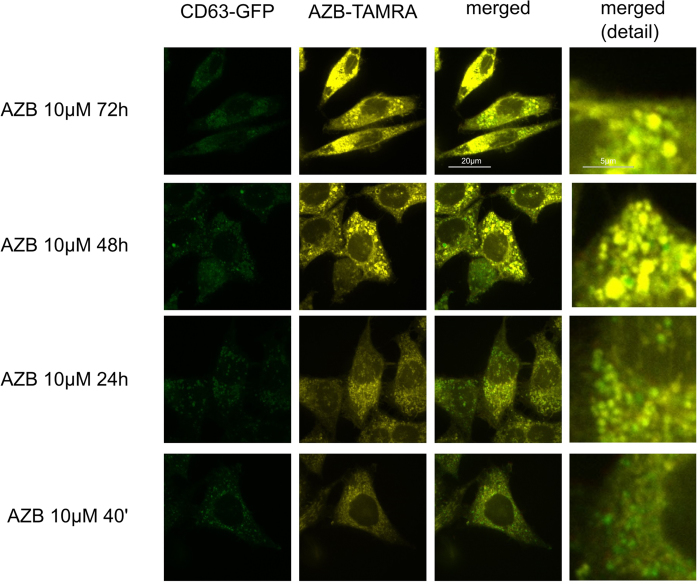
Colocalization of azidobupramine with the endo-lysosomal compartment. Hela cells stably expressing the endo-lysosomal marker CD63-GFP (green) were exposed to azidobupramine at 10 μM (5-TAMRA, yellow) and varying time periods (40 min, 24 hrs, 48 hrs, and 72 hrs) followed by UV-light crosslinking and click-chemistry; the colocalization of azidobupramine and CD63 becomes increasingly stronger over time; DMSO concentration was set to 0.1% in in all conditions.

**Figure 6:**
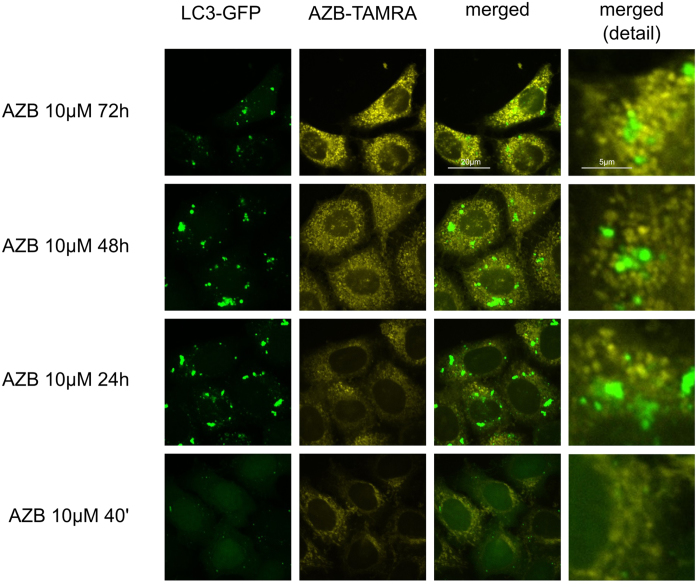
Colocalization of azidobupramine with the autophagosomal compartment. Hela cells stably expressing the autophagosomal marker LC3-GFP (green) were exposed to azidobupramine at 10 μM (5-TAMRA, yellow) and varying time periods (40 min, 24 hrs, 48 hrs, and 72 hrs) followed by UV-light crosslinking and click-chemistry; after 40 minutes at 10 μM azidobupramine hardly any LC3-clustering and colocalization of LC3 and azidobupramine can be observed; over time the LC3 clustering and the LC3/azidobupramine-colocalization becomes more prominent; DMSO concentration was set to 0.1% in in all conditions.

**Figure 7:**
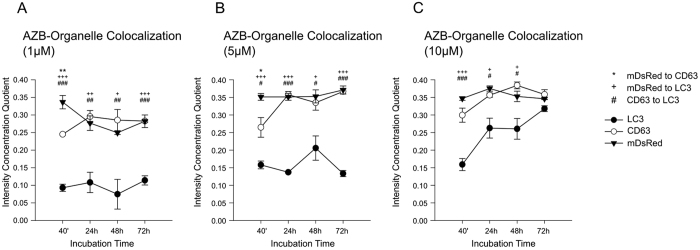
Analysis of concentration related effects on compartment specific distribution pattern. (**A**) *Preferred colocalization of azidobupramine with different compartments at 1 μM*: group analyses showed significant differences between both CD63 and mDsRED as compared to LC3 at all measurement times; moreover, the intensity correlation quotient for CD63 was lower than for mDsRED at 40 minutes (**A**). *Preferred colocalization of azidobupramine with different compartments at 5 μM*: the intensity correlation quotient was lower for LC3 as compared to both CD63 and mDsRED at all measurement times; moreover, at 40 minutes intensity correlation quotient for CD63 was lower than for mDsRED; throughout the experiment, time-related changes were observed for CD63 between 40 minutes and 24 hours as well as between 40 minutes and 72 hours (p = 0.011) (**B**). *Preferred colocalization of azidobupramine with different compartments at 10 μM*: group differences were observed between both mDsRED and CD63 as compared to LC3 at three first measurement times; throughout the protocol, time-related changes were observed for LC3 between 40 minutes and 24 hours (**C**). *p < 0.05; **p < 0.01; ***p < 0.001; each data point represents the mean (±SEM) of three independent experiments averaging the signal of 10 distinct cells; for more detailed statistical data please be referred to the supplementary material legend extension of Fig. 7.

First, compartment specific distribution patterns of azidobupramine were analyzed in relation to the three predefined compartments: the mitochondrial compartment (marker: mDsRed), the endo-lysosomal compartment (marker: CD63-GFP), and the autophagosomal compartment (marker: LC3-GFP) (Fig. 3A–C). The mitochondrial compartment was the first place of accumulation, independent of its applied concentration (Figs 4 and 3A; S-Fig. 5). Over time, however, the respective ICQs evolved differently not only in respect to the mitochondrial compartment but also to the endo-lysosomal (CD63-GFP) and autophagosomal (LC3-GFP) compartments. While the co-localization of mDsRed and azidobupramine remained stable at high concentrations (i.e. 5 μM and 10 μM), a significant decrease in ICQs was detected at 1 μM over time. The endo-lysosomal compartment, however, became significantly targeted between 40 minutes and 24 hours. Overall, our analyses revealed that endo-lysosomal vesicles positive for CD63-GFP are the most preferred places of azidobupramine accumulation (Figs 5 and 3B, S-Fig. 6). The most pronounced accumulation of azidobupramine within CD63 positive vesicles was found in cells that were stimulated with 10 μM azidobupramine over at least 48 hours (Figs 5 and 3B, S-Fig. 6). In contrast to the CD63 positive compartment, the LC3 compartment was targeted only at the highest concentration of azidobupramine (i.e. 10 μM) - most intensely after 72 hours (Figs 6 and 3C, S-Fig. 7).

Second, the intracellular distribution patterns of azidobupramine were analyzed in relation to the azidobupramine concentration applied (Fig. 7A–C). At 1 μM, the strongest colocalization was observed in the mitochondrial compartment and the CD63-positive endo-lysosomal compartment, while the LC3-positive autophagosomal compartment played only a subordinate role (Fig. 7A). The same applied to the 5 μM condition (Fig. 7B). At the 10 μM condition, however, the increasing colocalization of azidobupramine in the LC3-positive autophagosomal compartment became more apparent than in the mitochondrial and CD63-positive endo-lysosomal compartment (Fig. 7C).

When ICQs (Figs 3A–C and 7A-C) were put in relation to differences in intracellular accumulation of azidobupramine (Figs 1 and 2A), the extent of concentration and time-dependent differences became even more obvious. While azidobupramine signals were only low to moderate at 1 μM and 5 μM, concentrations of 10 μM resulted in an unexpected increase in signal intensity, which might have caused the rise in the ICQs of azidobupramine in the LC3-positive autophagosomal compartment. Further evaluation of the physiological implication of the increase in signal intensity and site specific colocalization, however, was not part of this study and should be the matter of future analyses.

### Analysis of LC3 and CD63 marker protein patterns

To put our findings in a broader context, we compared the effects of azidobupramine and the clinically active antidepressant clomipramine on CD63 vesicularity and LC3 clustering.

**Figure 8:**
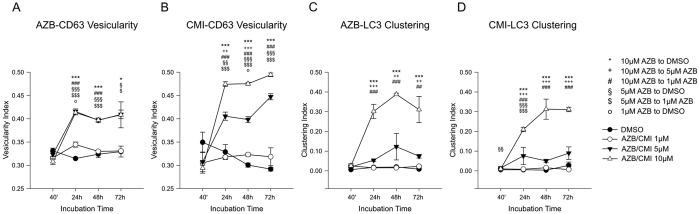
Comparison of the effects of azidobupramine and clomipramine on CD63-vesicularity and LC3-clustering. *Analysis of azidobupramine’s effects on CD63-vesicularity*: group analyses showed significant differences between DMSO and all AZB concentrations at 24 hours; moreover, at 24 hours AZB 1 μM showed a lower vesicularity index than both AZB 5 μM and AZB 10 μM; at 48 hours, both AZB 5 μM and AZB 10 μM produced a higher vesicularity index than both DMSO and AZB 1 μM; at 72 hours, DMSO showed a lower vesicularity index than both AZB 10 μM and AZB 5 μM, additionally vesicularity index for AZB 5 μM was higher than AZB 1 μM (**A**). *Analysis of clomipramine effects on CD63-vesicularity*: group analyses showed higher vesicularity for both CMI 10 μM and CMI 5 μM as compared to both DMSO and CMI 1 μM after 24 hours; moreover CMI 5 μM and CMI 10 μM differed in their vesicularity index at both 24 hrs and 48 hrs as well as CMI 1 μM was higher than DMSO at 48 hrs (**B**). *Analysis of azidobupramine effects on LC3-clustering*: group analyses showed a higher clustering index for AZB 10 μM as compared to AZB 5 μM, AZB 1 μM, and DMSO after 24 hours (**C**). *Analysis of clomipramine effects on LC3-clustering*: group analyses showed higher clustering index for CMI10 μM as compared to CMI5 μM, CMI1 μM, and DMSO after 24 h; moreover, CMI5 μM had higher clustering index than CMI1 μM at 24 h as well as DMSO both at 40’ and 24 h (**C**). *p < 0.05; **p < 0.01; ***p < 0.001; each data point represents the average of signal integration of 10 cells, each performed in triplicates; for more detailed statistical data please be referred to the supplementary material legend extension of Fig. 8.

CD63 vesicularity was equal to an increase of hollowness of CD63 positive vesicles (S-Fig. 3). The corresponding measure, i.e. the vesicularity index (VI), can adopt values between zero and one. The larger the VI, the more pronounced is the hollowness of CD63 positive vesicles (Fig. 8A,B).

At concentrations of 1 μM, azidobupramine and clomipramine exerted barely any effect on the VI of CD63 positive vesicles. However, a significant increase in VI was detected upon treatment with azidobupramine and clomipramine at 5 μM and 10 μM if the incubation time exceeded 40 minutes (Fig. 8A,B). It appears that 10 μM clomipramine stimulated the expansion of CD63 positive vesicles more intensely than 10 μM azidobupramine.

Lipidation and clustering of the LC3 protein has been frequently associated with CAD and antidepressant effects^30^,^31^,^32^. Therefore we compared the effects of DMSO, azidobupramine and clomipramine on LC3 clustering represented by the clustering index (CI). The CI is a measure indicative of the degree of signal condensation – high CI values is indicative to highly condensed dots and low CIs for a dispersed signal (S-Fig. 4). After DMSO treatment, we found the LC3 protein to be homogeneously dispersed throughout the cell which was reflected by CI values around zero (Fig. 8C,D). However, LC3-clustering occurred upon treatment with ≥ 5 μM azidobupramine or clomipramine. The highest CIs were achieved upon treatment with 10 μM azidobupramine or clomipramine for longer than 40 minutes (Fig. 8C,D).

## Discussion

Using azidobupramine as model substance, we demonstrate here for the first time that the vesicular accumulation of CADs is accompanied by an expansion of CD63-positive vesicles^33^,^34^. Furthermore, we show for the first time a time- and concentration-dependent intracellular accumulation of the CAD azidobupramine (Figs 1 and 2A) thereby further corroborating its suitability as a multifunctional tool for monitoring the intracellular fate of CADs. The substance concentrations chosen in the actual study were based on estimations of steady state concentrations of antidepressant in the central nervous system^35^,^36^, consensus guidelines for Therapeutic Drug Monitoring (TDM) in psychiatry^37^, and practiced reality. These considerations let us to include concentrations from 1 μM (therapeutic dose) to 10 μM (concentrations used in the cell biology and biochemical assays) enabling us to describe cellular effects at both non-toxic as well as at most likely toxic conditions.

Concentrations up to 5 μM azidobupramine led to an approximately linear increase in intracellular substance load over the total exposure time of 72 hours. A concentration of 10 μM, however, produced a disproportionate increase in intracellular substance load at later time points. A possible explanation for this phenomenon could be that limits of distinct saturable biological processes might be exceeded: Transport molecules such as the outward-facing P-glycoprotein (ABCB1), or other cellular detoxification processes are possible mechanisms that might be overrun at high CAD concentrations. Finally, we show that the effects of azidobupramine and the clinically active antidepressant clomipramine on CD63 vesicularity and LC3 clustering resemble each other underpinning azidobupramine as relevant tool to analyze intracellular distribution pattern of cationic amphiphilic drugs.

According to our findings, the mitochondrial compartment is one of the first intracellular target structures of azidobupramine (Fig. 7). This result is in accordance with findings of studies having used another analysis technique, namely a combination of radioactively labelled CADs together with differential centrifugation^7^,^8^,^9^,^10^. Theoretical considerations towards consequences of physicochemical properties of CADs on subcellular target structures resulted in hypotheses regarding their intracellular distribution pattern. According to these hypotheses and to measurements using radioactively labelled substances, CADs accumulate within the cytoplasm by a factor of 5–10 in comparison to the extracellular environment and further by a factor of 20–30 within the mitochondria in comparison to the cytoplasm. Depending on the test concentrations used, this led to the accumulation of CADs within mitochondria up to the millimolar range^38^. In addition to the electrochemical (ΔΨ) and pH gradients across outer membranes, also compartment-specific lipid compositions are held responsible for the mitochondrial accumulation^39^. In fact, twenty percent of the lipids of the inner membrane of the mitochondria are phospholipids known as facilitators of CAD translocation^39^,^40^,^41^.

According to literature, CADs initially become accumulated and protonated within the intermembrane space driven by ΔΨ, followed by a phospholipid mediated translocation step to the matrix and partial deprotonation within the matrix due to higher pH-values – the difference in pH values between intermembrane space and matrix is estimated to be 0.5^41^. This essentially means that once CADs become trapped within the mitochondria they are hindered from getting out of it. In our experiments, azidobupramine appeared to stably reside in the mitochondrial compartment at concentrations higher than 5 μM. At concentrations of 1 μM (Fig. 3A). However, azidobupramine showed strong colocalization with the mitochondrial compartment only at 40 minutes. While it is not possible to make statements on the actual concentrations of azidobupramine within the mitochondrial compartment, we conclude from our findings that a minimal amount of azidobupramine is necessary to keep azidobupramine constantly trapped within the mitochondria. In further studies, it remains to be clarified for each CAD whether and to what extent the mitochondrial accumulation is relevant for its clinical efficacy even though strong evidence exists to support the clinical relevance^42^,^43^.

In the case of azidobupramine, it was not the mitochondrial compartment, but certain vesicular structures that significantly account for the accumulation observed, especially at higher concentrations (Fig. 3A–C). In particular, vesicles that were positive for the CD63 marker were identified to be the most intensely targeted compartment (Fig. 3B). The CD63 surface-associated marker protein is involved in tightly regulated endocytotic and exocytotic processes, but also in other multiple intracellular cargo and trafficking activities. Of note, CD63-positive exosomes are also responsible for mRNA shuttling^44^. In a series of studies it has been shown that the CAD amiodarone induces an expansion of CD63-positive vesicles which were subsequently categorized as perinuclear hybrid-organelles sharing lysosomal and late-endosomal characteristics^14^. Similar effects on the enlargement of CD63-positive vesicles were also described for other CADs such as procainamide^45^. The study at hand, however, is the first that proves the colocalization of a CAD with CD63-positive vesicles and that monitors the CAD accumulation within this compartment over time and at different concentrations.

The origin of the CD63-positive vesicles has been discussed quite diversely. While some studies propose that CD63-positive vesicles originate from the trans-Golgi apparatus, others suggest that mitochondria are involved in their formation^45^. CAD-induced changes of the CD63-associated endocytotic and exocytotic pathways may account for the expansion and accumulation of CD63-positive vesicles overloaded with CADs.

The LC3 lipoprotein is strongly linked to autophagy and autophagy-associated processes^46^. Furthermore, it is well known that CADs modulate autophagy and autophagy-associated processes and intervene with LC3 and LC3 metabolism^14^. We show here that colocalization of LC3 and azidobupramine occurred only at concentrations of 10 μM and exposure times of at least 24 hours (Fig. 3C). It is not clear at this point whether this colocalization is of physiological relevance or a mere reflection of the massive intracellular overload of azidobupramine.

In summary, this study shows that azidobupramine is a substance that allows for studying intracellular CAD distribution pattern. We illustrate that the subcellular localization of azidobupramine can be traced in a way that allows temporal and spatial resolution. Furthermore, we demonstrate that the distribution of azidobupramine follows a characteristic pattern that is strongly dependent on the substance concentrations and exposure times. After reaching the mitochondrial compartment, azidobupramine, in particular at higher concentrations, seemed to be (re-)distributed to CD-63 positive vesicles and LC3 compartments (Fig. 7C). It is important for further studies that a minimum amount of azidobupramine is necessary to initiate accumulation. While only weak but significant signals could be monitored after cells were treated at 1 μM azidobupramine, strong accumulation was observed only after the treatment at 10 μM.

Future studies have to be conducted to explore how the herein observed intracellular CAD distribution pattern is related to CAD function. The findings here help to further elucidate the intracellular functions of CADs and in particular of antidepressants. Such knowledge is essential since other mechanisms of action than enhancement of monoaminergic neurotransmission may play an important role in conveying beneficial clinical effects^47^,^48^,^49^,^50^,^51^,^52^,^53^,^54^.

## Methods

### Chemical Synthesis

Azidobupramine was synthesized as described recently^28^.

### Materials and Chemicals

The following materials were purchased: Minimal Essential Media (MEM), FreeStyleTM 293 Expression medium, Fetal Bovine Serum (FBS), antibiotic-antimycotic, L-glutamine, and non-essential amino acids, Alexa-Fluor-488-Azide, Prolong Gold Antifade, WGA Alexa-Fluor-647-Azide conjugate (*Life Technologies, CA, USA*); clomipramine, Hank’s Balanced Salt Solution (HBSS), gelatin (*SigmaAldrich, MO, USA*); PFA 37% (*Merck, Darmstadt, Germany*); BTTAA was kindly provided by S. Sieber (*Technical University Munich, IAS, CIPSM, Department of Chemistry, Garching, Germany*).

### Cell culture

HeLa cells stably expressing EGFP-CD63, EGFP-LC3, and mDsRed were kindly provided by A.M. Tolkovski (*Department of Biochemistry, University of Cambridge, Cambridge, UK*)^55^; for immunohistochemical analysis, cells were cultivated adherently (90% MEM, 10% FBS, 2 mM L-glutamine, non-essential amino acids).

### Stimulation experiments

For stimulation experiments, HeLa cells were cultivated on glass plates precoated with 0.1% gelatin, inoculated at a cell density of approximately 2500 cells/cm^2^. After a resting period of 12 hours, cells were kept in cultivation over another 72 hours without changing the medium. Cells were stimulated over different time periods (72 hrs, 48 hrs, 24 hrs, and 40 min) and at different concentrations (10 μM, 5 μM, and 1 μM) of clomipramine or azidobupramine. The stock solutions for clomipramine and azidobupramine were prepared in DMSO at a 1000 fold higher concentrations than necessary for the stimulation (10 mM, 5 mM, and 1 mM); the dilution guarantees that the final DMSO concentration does not exceed 0.1%. For control conditions, only the vehicle DMSO was used.

### Histochemical staining

For histochemical staining, cells were exposed to UV-light using the Dual Transilluminator from Stratagene (*5* × *8 Watt at 312 nm, La Jolla, CA, USA*) for 60 seconds. Then, paraformaldehyde (PFA) was added to the medium to a final concentration of 4% over 30 minutes at cell culture conditions (37 °C, 5% CO_2_). This was followed by three washing steps with HBSS, a cell membrane staining process (1 μg/ml WGA647 diluted in HBSS, 7’, RT), and an additional fixation step with 4% PFA (30′, RT). To prepare cells for the copper mediated click reaction, cells were washed three times with PBS (RT) followed by the actual CuAAC reaction conducted for 30 minutes at RT in PBS supplemented with 25 μM fluorophore-azide, 2.5 mM ascorbic acid, 250 μM CuSO4, and 500 μM bis[(tertbutyltriazoyl)methyl]-[(2-carboxymethyltriazoyl)methyl]amine (BTTAA) (S-Fig. 1). The fluorescent dyes used were 5-TAMRA-Azide (*baseClick, Neuried, Germany*) for HeLa cells stably expressing EGFP-CD63 or EGFP-LC3, and Alexa-Fluor-488-Azide for HeLa cells positive for mDsRed (S-Fig. 1); the fluorophores Alexa-Fluor-647, Alexa-Fluor-488-Azide, and 5-TAMRA-Azide were used because of their known stability in a broad range of pH values. After three further washing steps with PBS, the coverslips were immersed into water before they were embedded in ProLong Gold Antifade. The specimens were kept at dark and cold (4 °C) until analysis.

### Microscopy

Microscopic images were acquired by the commercially available Zeiss Cell Observer SD with a Yokogawa spinning disk unit. To account for different experimental setups (i.e. quantification of azidobupramine, determination of CD63-vesicularity, and measurement of LC3-clustering), laser powers and exposure settings were adjusted individually and kept constant throughout the whole measurement allowing comparability. For the analysis of LC3- and CD63-topology and for colocalization measurements, single slices through the lower part of the cells with an optical section depth of 270 nm were recorded. For uptake measurements, z-stacks from the bottom to the top of the cells were acquired with a z-spacing of 270 nm.

### Image analysis

For data analysis and visualization, Fiji^56^ and Imaris (Bitplane AG, Zurich, Switzerland) were employed. For uptake measurements, background fluorescence was subtracted at all slices of the z-stack. Then, a threshold was applied in order to quantify only the internalized AZB signal. Finally, the intracellular AZB signal was summed up over all slices. The result was defined as the amount of internalized AZB. For colocalization measurements and analysis of LC3- and CD63-topology, special algorithms were applied that are described in detail in the supplementary material (S-Figs 2, 3 and 4). Briefly, for colocalization analysis, Li’s ICQ method was applied^29^ (S-Fig. 2). It compares pixel-wise, after normalization of the image histograms, whether the intensities of the two investigated images change synchronously (indicating colocalization), randomly (indicating random colocalization) or asynchronously (indicating anticolocalization). This analysis yields the colocalization coefficient, ranging from −0.5 (complete anticolocalization) over 0 (random colocalization) to + 0.5 (complete colocalization)^29^. For the analysis of CD63, we defined the term solidity of the CD63-signals as explained in the supplementary material (S-Fig. 3). The term solidity was defined as quotient between the area of the fluorescence signal and that area of the convex hull. The lower this value is, the less solid and the hollower (i.e. vesicle-like) is the signal. Vesicularity then is defined as one minus solidity thus ranging from zero to one. Finally, for clustering analysis of LC3, the total amount of the LC3-signal was quantified in a first step (S-Fig. 4). Then, we defined a threshold in such a way that only the LC3-signal localized in clusters was quantified. The ratio of the LC3-signal in clusters divided by the total LC3-signal was defined as the cluster index, ranging from zero to one.

### Statistical analyses

Statistical analyses were performed with SPSS software version 18.0 (SPSS Inc., Chicago IL). Analysis of the intracellular azidobupramine signal by means of fluorescence microscopy (Fig. 2A), analysis of colocalisation of azidobupramine with the mitochondria compartment or vesicular compartment positive for CD-63 and LC3 (Figs 6A–C and 7A–C), analysis of the CD-63 vesicularity index (Fig. 8A,B), and analysis of the LC3 clustering index (Fig. 8C,D) were calculated with two-way ANOVAs with repeated measures. In case of violation of the assumption of sphericity, Greenhouse-Geisser corrections were applied. Analysis of the control experiments for the fluorescence signal (Fig. 2B) was performed with one-way ANOVA. Multiple comparisons were Bonferroni corrected. All data are shown as mean ± standard error of measurement (SEM). The significance level was set at p ≤ .05.

## Additional Information

**How to cite this article:** Vater, M. *et al*. New insights into the intracellular distribution pattern of cationic amphiphilic drugs. *Sci. Rep.* **7**, 44277; doi: 10.1038/srep44277 (2017).

**Publisher's note:** Springer Nature remains neutral with regard to jurisdictional claims in published maps and institutional affiliations.

## Supplementary Material

Supplementary Information
