# Corrigendum: New insights into the intracellular distribution pattern of cationic amphiphilic drugs

**DOI:** 10.1038/srep46011

**Published:** 2017-04-06

**Authors:** Magdalena Vater, Leonhard Möckl, Vanessa Gormanns, Carsten Schultz Fademrecht, Anna M. Mallmann, Karolina Ziegart-Sadowska, Monika Zaba, Marie L. Frevert, Christoph Bräuchle, Florian Holsboer, Theo Rein, Ulrike Schmidt, Thomas Kirmeier

Scientific Reports 7: Article number: 44277; 10.1038/srep44277 published online: 03 10 2017; updated: 04 06 2017.

The Acknowledgements section in this Article is incomplete.

“NeuroNova gGmbH, Munich provided financial support between 2011 and 2016 aiming at *The investigation of novel molecular mechanisms of antidepressants at cellular level*”.

should read:

“NeuroNova gGmbH, Munich provided financial support between 2011 and 2016 aiming at *The investigation of novel molecular mechanisms of antidepressants at cellular level.* C.B. and L.M. thank the excellence clusters Nanosystems Initiative Munich (NIM) and the Center for Integrated Protein Science Munich (CIPSM) as well as the Center for Nanoscience Munich (CeNS)”.

