# Supplementary material for: New insights into the intracellular distribution pattern of cationic amphiphilic drugs
Source: Sci Rep. 2017 Mar 10;7:44277. doi: 10.1038/srep44277 (PMC5345070; doi:10.1038/srep44277)
Supplement: Supplementary Information [file srep44277-s1.pdf]

Supplementary Information

New insights into the intracellular distribution pattern of cationic amphiphilic drugs

Magdalena Vater, Leonhard Möckl, Vanessa Gormanns, Carsten Schultz Fademrecht, Anna M. Mallmann, Karolina Ziegart-Sadowska, Monika Zaba, Marie L. Frevert, Christoph Bräuchle, Florian Holsboer, Theo Rein, Ulrike Schmidt and Thomas Kirmeier

S-Figure 1: Principle azidobupramine target interaction

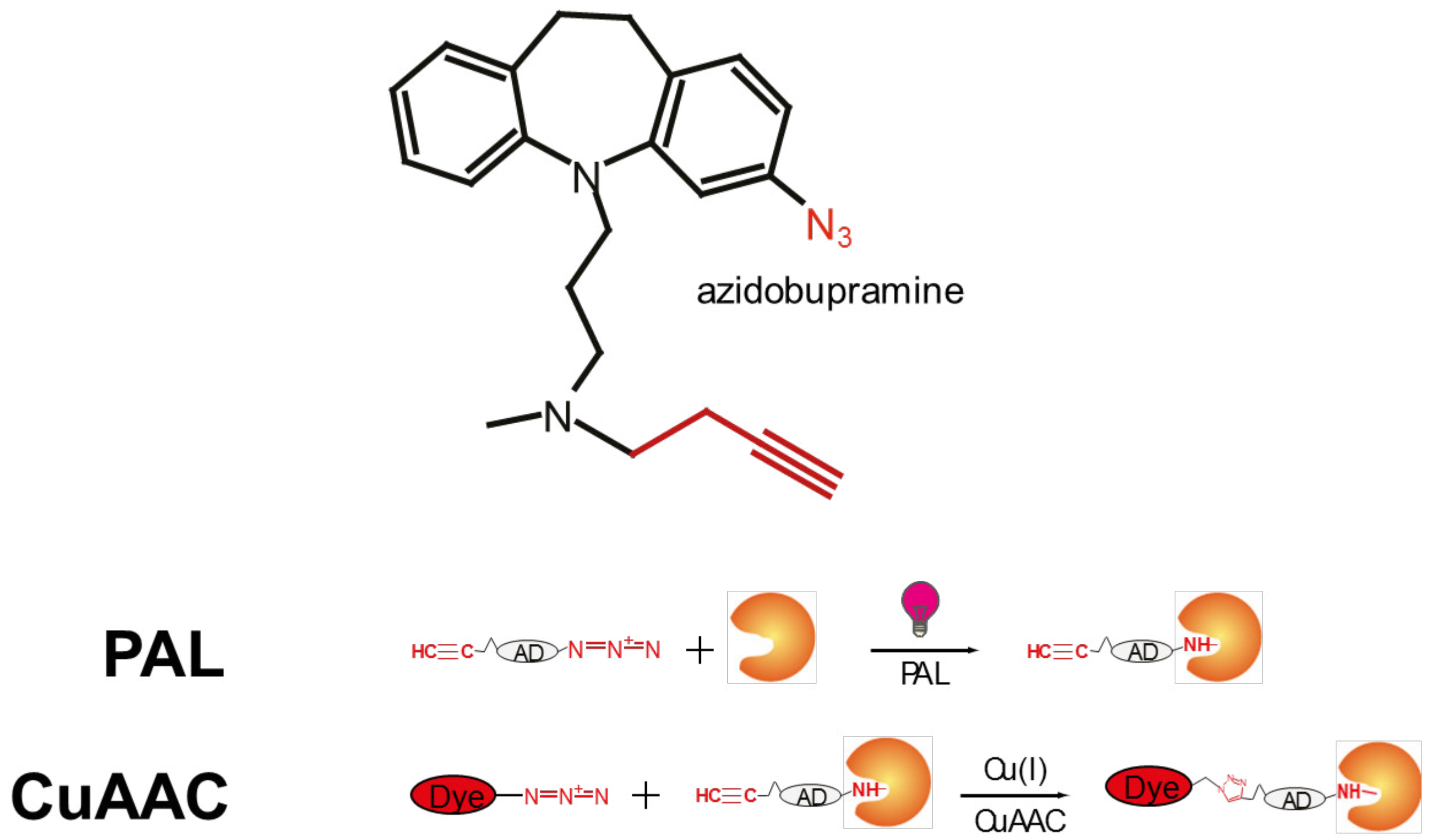

S-Figure 1 Principle azidobupramine target interaction. Top: Azidobupramine is characterized by an additional azide-group in the aromatic head structure amenable for Phoroaffinity labeling (PAL) and an alkyne-group at the terminal amino-group amenable for copper mediated click reaction (CuAAC). Bottom: During the process of PAL the aryl-azide group becomes activated by means of UV-light resulting in an insertion of the reactive nitrene-group into C-H and N-H bonds; CuAAC is used to label azidobupramine-target protein complexes with fluorophores.

S-Figure 2: Principle of the ICQ analysis

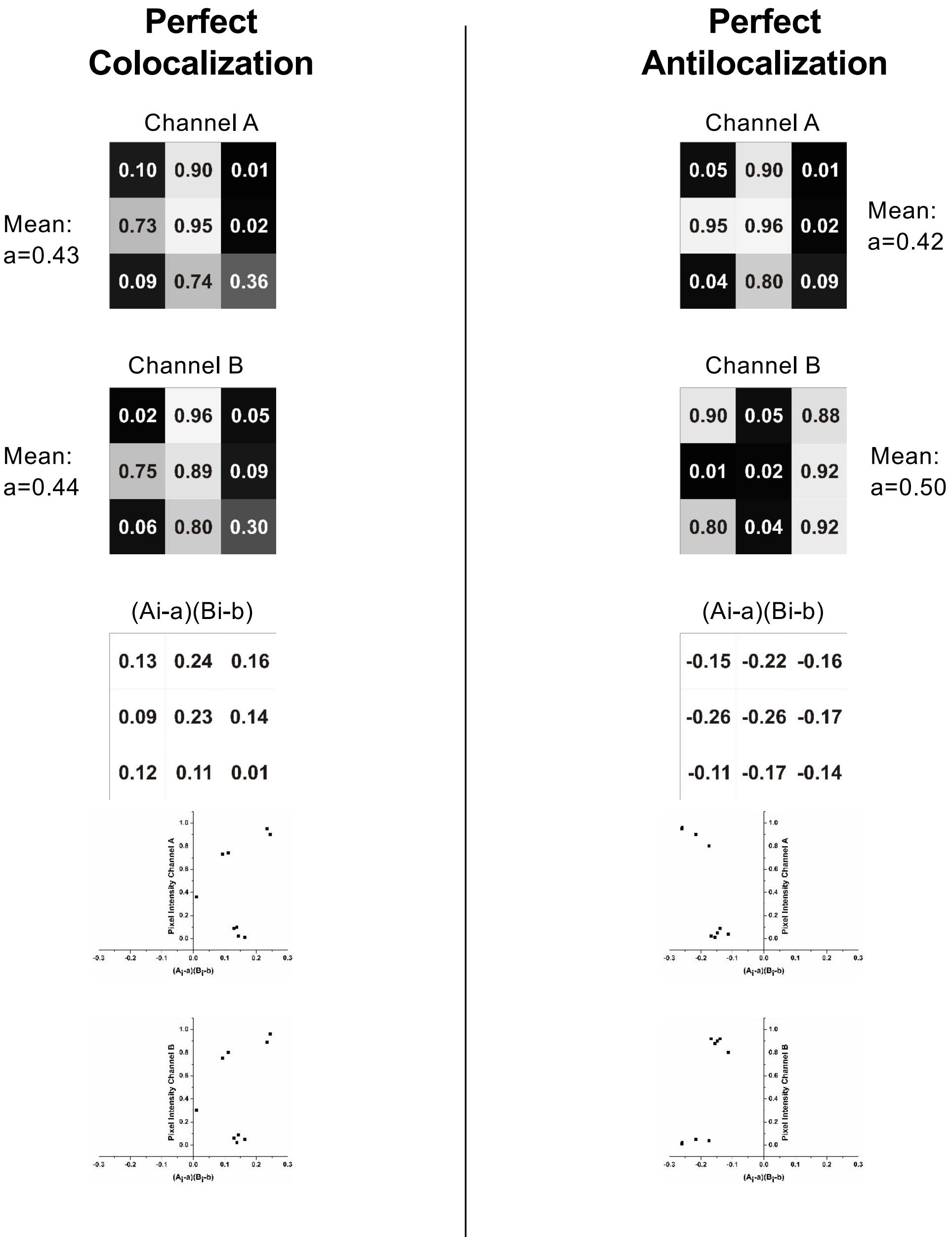

$$ICQ = \frac{(PositiveProducts)}{(Products)} - 0.5 = 9/9 - 0.5 = 0.5$$

$$ICQ = \frac{(PositiveProducts)}{(Products)} - 0.5 = 0/9 - 0.5 = -0.5$$

S-Figure 2 Principle of the ICQ analysis. Left column: Perfect colocalization. Right column: Perfect anticolocalization. An example image of 3x3 pixels is shown. In case of perfect colocalization between channel A and B, the intensities of a pixel in channel A (A1, A2, …, An) and the equivalent pixel in channel B (B1, B2, …, Bn) change synchronously – a high/low intensity of a pixel in one channel corresponds to a high/low intensity in the other channel. In case of anticolocalization, a high pixel intensity in one channel corresponds to a low pixel intensity in the other and vice versa. To calculate the ICQ, the mean of all pixels (a, b) in one channel is determined. Then, for each pixel, the mean is subtracted and the product of the resulting values of the corresponding pixels in channel A and B is calculated, i.e. (Ai-a)(Bi-b). If the intensity of each pixel is plotted against the product, colocalization is visible from a curved line in the positive product values, whereas anticolocalization is visible from a curved line in the negative product values. The ICQ is the quotient between positive products and the numbers of products (i.e. the number of pixels) minus 0.5; 0.5 is just subtracted for convenience to yield values between -0.5 and 0.5.

S-Figure 3: Analysis of CD-63 vesicularity

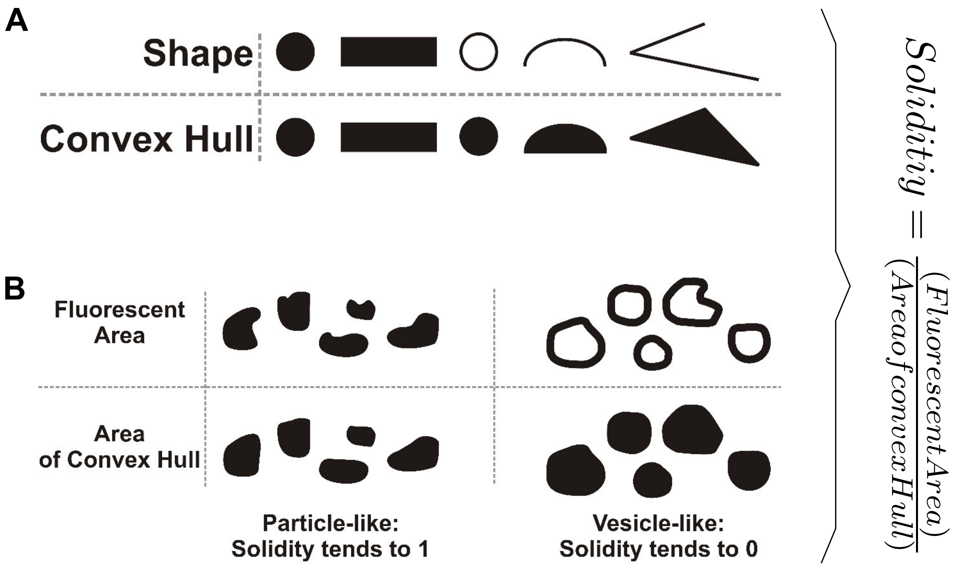

S-Figure 3 Analysis of CD-63 vesicularity. For the vesicularity analysis the convex hull of a shape was defined in a first step; the convex hull of a number of points is the smallest set that contains all points (A). In the next step, the convex hull was used to discriminate between particle-like and vesicle-like fluorescence signals; the quotient between the fluorescent area and the area of the convex hull is called solidity, which tends to one if the structures are particle-like and to zero if the structures are vesicle-like; in our study we defined one minus the solidity as the vesicularity index (VI) of the fluorescence signal (B).

S-Figure 4: Analysis of LC3-Clustering

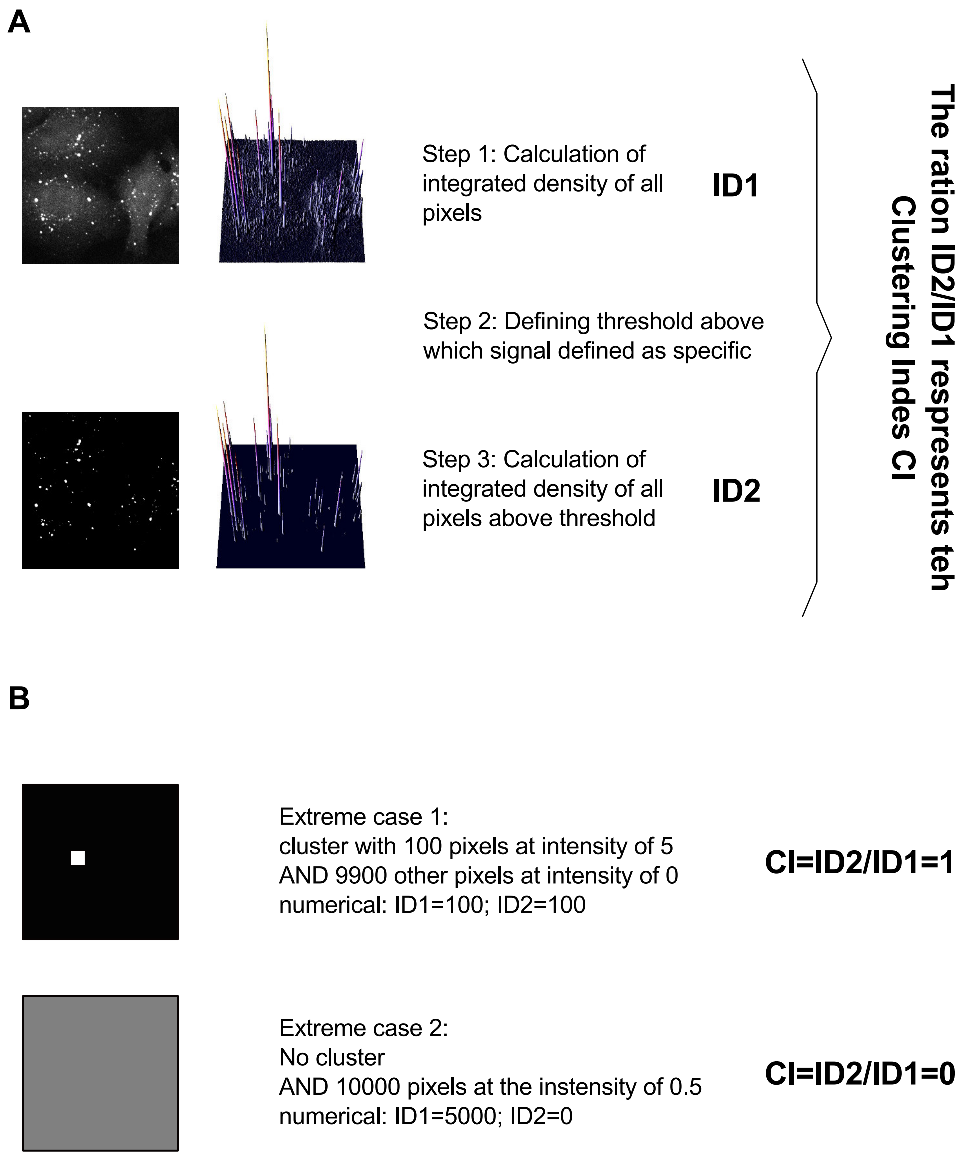

S-Figure 4 Analyiss of LC3-Clustering. The clustering analysis followed a multistage evaluation step: In the first step, the intensity of all pixels was calculated (ID1). This was followed by step two applying a threshold to eliminate the background signal. In the third step the integrated density of all pixels was calculated (ID2); the ratio ID2/ID1 was defined as clustering index (CI) (A). The second part of the figure describes two example cases describing the two possible extreme cases (B).

S-Figure 5: Colocalization of azidobupramine with mDsRed

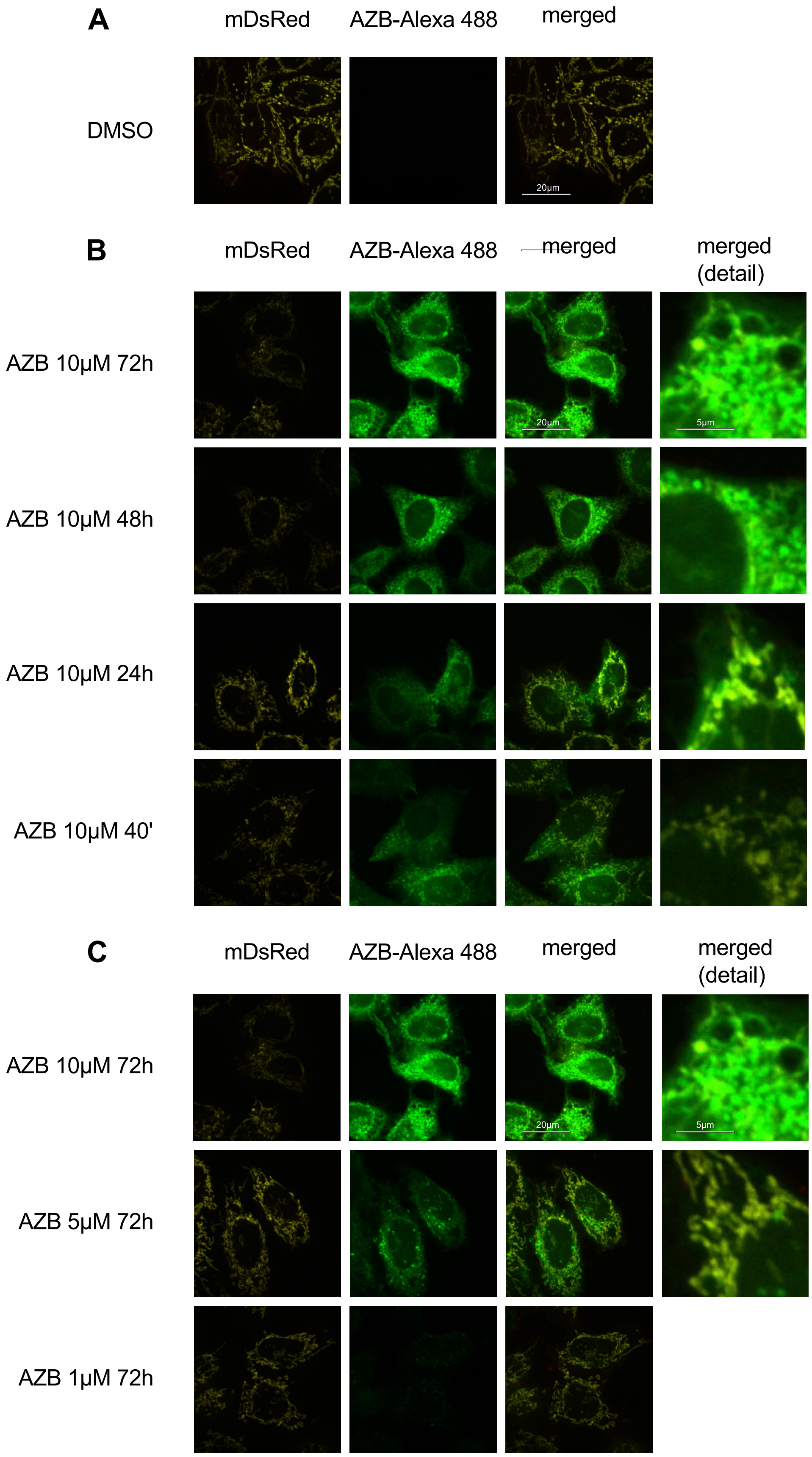

S-Figure 5 Colocalization of azidobupramine with the mitochondrial compartment. Hela cells stably expressing mDsRed, a marker protein (red) for the mitochondrial compartment, were exposed to azidobupramine followed by UV-light crosslinking and click-chemistry; negative control using DMSO for treatment (A); comparison of differences in distribution pattern of azidobupramine after cell treatment with 10µM at four different time points (40 min , 24 hrs, 48 hrs, and 72 hrs) (B);comparison of differences in distribution pattern of azidobupramine after cell treatment at three different concentrations (i.e. 1 µM, 5 µM, and 10 µM) over a treatment duration of 72 hrs (C).

S-Figure 6: Colocalization of azidobupramine with CD63-GFP

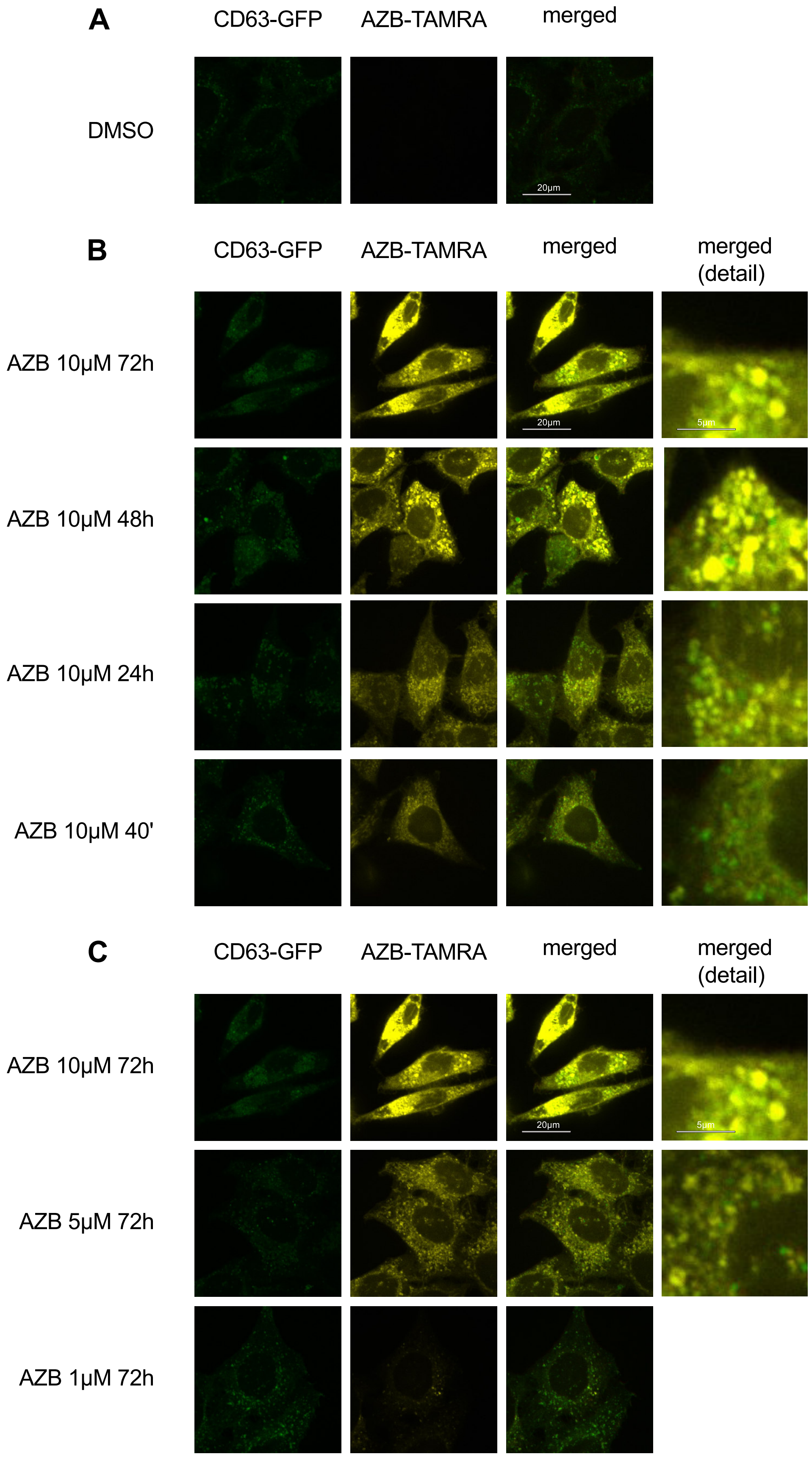

S-Figure 6 Colocalization of azidobupramine with the endo-lysosomal compartment. Hela cells stably expressing the endo-lysosomal marker CD63-GFP (green) were exposed to azidobupramine followed by UV-light crosslinking and click-chemistry; negative control using DMSO for treatment (A); comparison of differences in distribution pattern of azidobupramine after cell treatment with 10µM at four different time points (40 min , 24 hrs, 48 hrs, and 72 hrs) (B);comparison of differences in distribution pattern of azidobupramine after cell treatment at three different concentrations (i.e. 1 µM, 5 µM, and 10 µM) over a treatment duration of 72 hrs (C).

S-Figure 7: Colocalization of azidobupramine with LC3-GFP

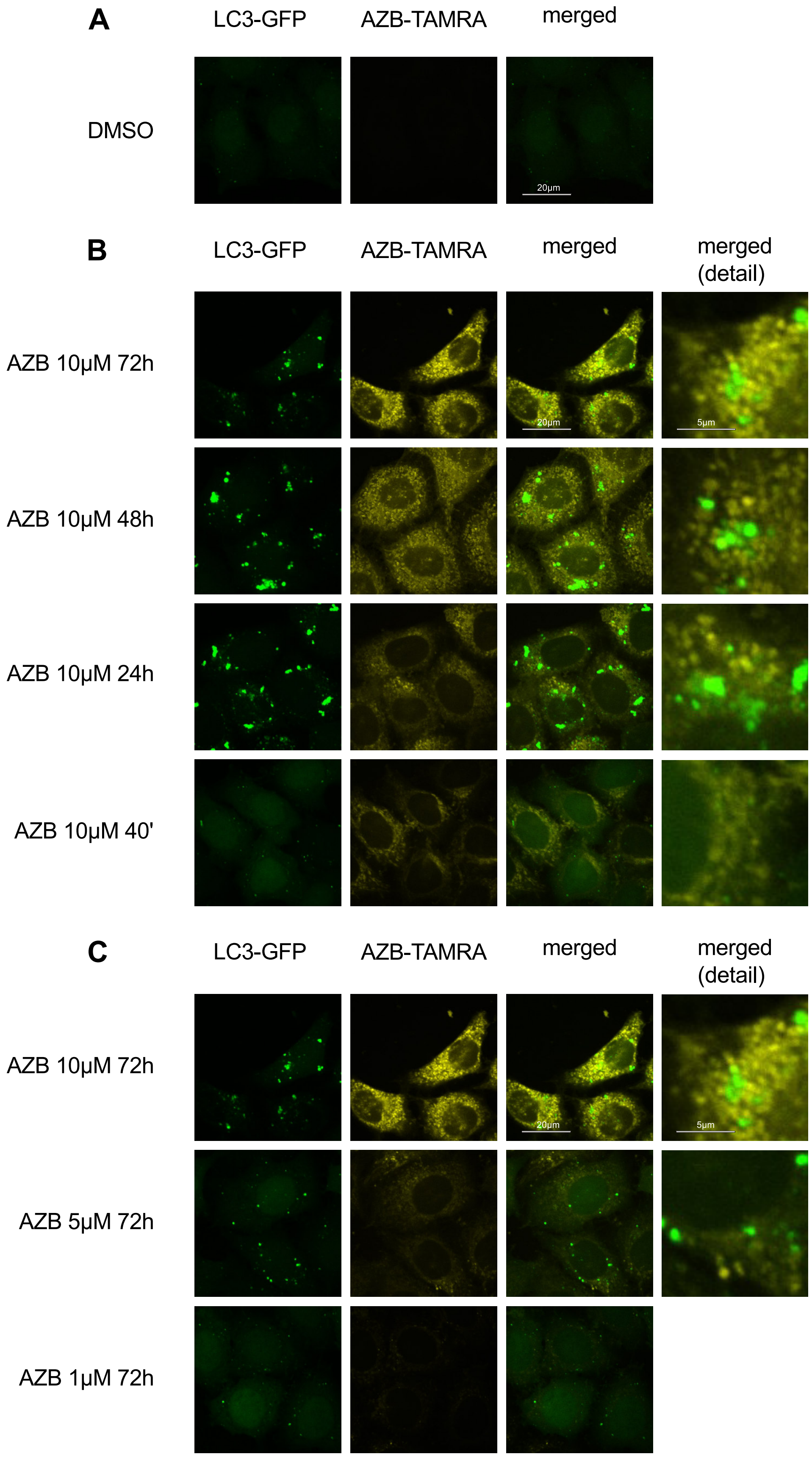

S-Figure 7 Colocalization of azidobupramine with the autophagosomal compartment. Hela cells stably expressing the autophagosomal marker LC3-GFP (green) were exposed to azidobupramine followed by UV-light crosslinking and click-chemistry; negative control using DMSO for treatment (A); comparison of differences in distribution pattern of azidobupramine after cell treatment with 10µM at four different time points (40 min , 24 hrs, 48 hrs, and 72 hrs) (B);comparison of differences in distribution pattern of azidobupramine after cell treatment at three different concentrations (i.e. 1 µM, 5 µM, and 10 µM) over a treatment duration of 72 hrs (C).

Supplementary Figure legends

Quantification of intracellular azidobupramine (legend extension of Figure 2 in the manuscript). *Quantification of the azidobupramine specific intracellular fluorescence signal at Hela cells; each data point represents the mean (±SEM) of three independent experiments comprising the integration and averaging of the fluorescent signal of 10 distinct cells*: we found significant main effects of group ($F(3, 8) = 25.90$, $p \leq .001$) and time ($F(3, 1.04) = 9.89$, $p = .013$) as well as an interaction effect ($F(9, 3.11) = 8.28$, $p = .007$); post-hoc group differences (Bonferroni corrected) revealed higher cellular fluorescence under AZB 10 µM as compared to DMSO at all measurement times (40’, p = .05; 24 hrs, p = .018; 48 hrs, p ≤ .001; 72 hrs, p = .008), as compared to AZB 1 µM after 24 hours (24 hrs, p = .023; 48 hrs, p ≤ .001; 72 hrs, p = .008), and as compared to AZB 5 µM after 48 hours (48 hrs, p ≤ .001; 72 hrs, p = .014); throughout the assessment, cellular fluorescence intensity became higher only under AZB 10 µM concentration (between 40 min and 48 hrs, p ≤ .001; between 40 min and 72 hrs, p = .001; between 24 hrs and 48 hrs, p ≤ .001; between 24 hrs and 72 hrs, p = .002; between 48 hrs and 72 hrs, p = .019) **(A)**.

Analysis of compartment specific distribution pattern of azidobupramine related to concentrations used (legend extension of Figure 3 in the manuscript): *Colocalization of azidobupramine with the mitochondrial compartment was analyzed with two-way ANOVA with repeated measures; each data point represents the mean (±SEM) of three independent experiments averaging the signal of 10 distinct cells*: we found significant main effect of group ($F(2, 6) = 58.26$, $p \leq .001$) and an interaction effect ($F(6) = 3.13$, $p = .028$); after 24 hours, the intensity correlation quotient was lower under AZB 1 µM in comparison to AZB 5 µM as well as to AZB 10 µM (AZB 1 µM vs. AZB 5 µM: 24 hrs, p = .027; 48 hrs, p = .006; 72 hrs, p .007; AZB 1 µM vs. AZB 10 µM: 24 hrs: p = .007; 48 hrs: p = .006; 72 hrs: p = .033); throughout the assessment, time-related changes were observed only under AZB 1 µM (between 40 min

and 24 hrs, p = .027; between 40 min and 48 hrs, p = .018) **(A)**. *Colocalization of azidobupramine with the endo-lysosomal compartment was analyzed with two-way ANOVA with repeated measures; each data point represents the mean (±SEM) of three independent experiments averaging the signal of 10 distinct cells*: we found main effects of group ($F(2, 6) = 31.82$, $p = .001$) and time ($F(3) = 10.60$, $p \leq .001$); post-hoc analyses (Bonferroni corrected) revealed at both measurement times 24 hours and 72 hours significant differences between AZB1µM as compared with AZB5µM as well as with AZB10µM (AZB 1 µM vs. AZB 5 µM: 24 hrs, p = .028; 72 hrs, p = .002; AZB 1 µM vs. AZB 10 µM: 24 hrs, p = .031; 72 hrs, p = .004); throughout the experiment, time-related changes were observed only under AZB 5 µM between 40 minutes and 72 hours (p = .016) **(B)**. *Colocalization of azidobupramine with the autophagosomal compartment was analyzed with two-way ANOVA with repeated measures; each data point represents the mean (±SEM) of three independent experiments averaging the signal of 10 distinct cells*: we found significant main effect of group ($F(2, 6) = 36.84$, $p \leq .001$) and an interaction effect ($F(6, 2.50) = 4.35$, $p = .05$). Post-hoc group differences (Bonferroni corrected) were observed between AZB 1 µM and AZB 10 µM at all measurement times (40 min, p = .037; 24 hrs, p = .01; 48 hrs, p = .032; 72 hrs, $p \leq .001$); moreover, intensity correlation quotient for AZB 1 µM was lower than AZB 5 µM at 40 minutes (p = .039) and for AZB 5 µM was lower than AZB 10 µM at both 24 hours (p = .027) and 72 hours ($p \leq .001$); throughout the experiment, time-related differences were observed only under AZB 10 µM between 40 minutes and 24 hours (p = .024) as well as between 40 minutes and 72 hours ($p \leq .001$) **(C)**.

Analysis of concentration related effects on compartment specific distribution pattern (legend extension of Figure 7 in the manuscript): (A) *Preferred colocalization of azidobupramine with different compartments at 1µM;_each data point represents the mean (±SEM) of three independent experiments averaging the signal of 10 distinct cells*: Two-way

ANOVA with repeated measures revealed (mean±SEM) main effect of group ($F(2, 6) = 92.95$, $p \leq .001$); post-hoc group analyses (Bonferroni corrected) showed significant differences between both CD63 and mDsRED as compared to LC3 at all measurement times (LC3 vs. CD63: 40 min, $p \leq .001$; 24 hours, $p = .003$; 48 hours, $p = .008$; 72 hours, $p \leq .001$; LC3 vs. mDsRED: 40 min, $p \leq .001$; 24 hours, $p = .006$; 48 hours, $p = .019$; 72 hours, $p \leq .001$); moreover, the intensity correlation quotient for CD63 was lower than for mDsRED at 40 minutes ($p = .006$) **(A)**. *Preferred colocalization of azidobupramine with different compartments at 5µM; each data point represents the mean (±SEM) of three independent experiments averaging the signal of 10 distinct cells*: Two-way ANOVA with repeated measures revealed (mean±SEM) main effect of group ($F(2, 6) = 163.27$, $p \leq .001$) and an interaction effect ($F(6) = 4.26$, $p = .008$); the intensity correlation quotient was lower for LC3 as compared to both CD63 and mDsRED at all measurement times (LC3 vs. CD63: 40 min, $p = .018$; 24 hrs, $p \leq .001$; 48 hrs, $p = .038$; 72 hrs, $p \leq .001$; LC3 vs. mDsRED: 40 min, $p = .001$; 24 hrs, $p \leq .001$; 48 hrs, $p = .021$; 72 hrs, $p \leq .001$); moreover, at 40 minutes intensity correlation quotient for CD63 was lower than for mDsRED ($p = .045$); throughout the experiment, time-related changes were observed for CD63 between 40 minutes and 24 hours ($p = .032$) as well as between 40 minutes and 72 hours ($p = .011$) **(B)**. *Preferred colocalization of azidobupramine with different compartments at 10µM; each data point represents the mean (±SEM) of three independent experiments averaging the signal of 10 distinct cells*: two-way ANOVA with repeated measures revealed significant main effects of group ($F(2, 6) = 90.19$, $p \leq .001$) and time ($F(3) = 11.67$, $p \leq .001$) as well as an interaction effect ($F(6) = 4.32$, $p = .007$); post-hoc group differences (Bonferroni corrected) were observed between both mDsRED and CD63 as compared to LC3 at three first measurement times (mDsRED vs. LC3: 40 min, $p \leq .001$; 24 hrs, $p = .011$; 48 hrs, $p = .05$; CD63 vs. LC3: 40 min, $p = .002$; 24 hrs, $p = .025$; 48 hrs, $p = .014$);

throughout the protocol, time-related changes were observed for LC3 between 40 minutes and 24 hours ($p = .012$) as well as between 40 minutes and 72 hours ($p = .001$) **(C)**.

Comparison of the effects of azidobupramine and clomipramine on CD63-vesicularity and LC3-clustering (legend extension of Figure 8 in the manuscript): *Analysis of azidobupramine's effects on CD63-vesicularity; each data point represents the mean (±SEM) of three independent experiments averaging the signal of 10 distinct cells*: two-way ANOVA with repeated measures revealed main effects of group ($F(3, 8) = 64.59$, $p \leq .001$) and time ($F(3, 1.31) = 28.07$, $p \leq .001$), as well as an interaction effect ($F(9, 3.92) = 8.07$, $p = .003$); post-hoc group differences (Bonferroni corrected) were observed between DMSO and all AZB concentrations at 24 hours (DMSO vs. AZB 1 µM: $p = .011$; DMSO vs. AZB 5 µM: $p \leq .001$; DMSO vs. AZB 10 µM: $p \leq .001$); moreover, at 24 hours AZB 1 µM showed a lower vesicularity index than both AZB 5 µM ($p \leq .001$) and AZB 10 µM ($p \leq .001$); at 48 hours, both AZB 5 µM and AZB 10 µM produced a higher vesicularity index than both DMSO and AZB 1 µM (AZB 5 µM vs. DMSO: $p \leq .001$; AZB 5 µM vs. AZB 1 µM: $p \leq .001$; AZB 10 µM vs. DMSO: $p \leq .001$; AZB 10 µM vs. AZB 1 µM: $p \leq .001$); at 72 hours, DMSO showed a lower vesicularity index than both AZB 10 µM ($p = .047$) and AZB 5 µM ($p = .044$), additionally vesicularity index for AZB 5 µM was higher than AZB 1 µM ($p = .048$); throughout the experiment, time-dependent differences were observable for AZB 10 µM (between 40 min and 24 hrs: $p \leq .001$; between 40 min and 48 hrs: $p \leq .001$; between 40 min and 72 hrs: $p = .005$), AZB 5 µM (between 40 min and 24 hrs: $p \leq .001$; between 40 min and 48 hrs: $p \leq .001$; between 40 min and 72 hrs, $p = .013$; between 24 hrs and 48 hrs: $p = .048$), and AZB 1 µM (between 40 min and 24 hrs: $p = .027$) **(A)**. *Analysis of clomipramine effects on CD63-vesicularity; each data point represents the mean (±SEM) of three independent experiments averaging the signal of 10 distinct cells*: Two-way ANOVA with repeated measures revealed main effects of group ($F(3, 8) = 160.69$, $p \leq .001$) and time ($F(3) = 34.15$, p

$\leq .001$), as well as an interaction effect ($F(9) = 20.66$, $p \leq .001$); post-hoc group differences (Bonferroni corrected) showed higher vesicularity for both CMI 10 µM and CMI 5 µM as compared to both DMSO and CMI 1 µM after 24 hours (CMI 10 µM vs. DMSO: 24 hrs, $p \leq .001$; 48 hrs: $p \leq .001$; 72 hrs: $p \leq .001$; CMI 5 µM vs. DMSO: 24 hrs, $p = .003$; 48 hrs, $p \leq .001$; 72 hrs, $p \leq .001$; CMI 10 µM vs. CMI 1 µM: 24 hrs, $p \leq .001$; 48 hrs, $p \leq .001$; 72 hrs, $p \leq .001$; CMI 5 µM vs. CMI 1 µM: 24 hrs, $p = .001$; 48 hrs, $p \leq .001$; 72 hrs: $p \leq .001$); moreover CMI 5 µM and CMI 10 µM differed in their vesicularity index at both 24 hrs ($p = .006$) and 48 hrs ($p \leq .001$) as well as CMI 1 µM was higher than DMSO at 48 hrs ($p = .05$); time differences were shown for CMI 10 µM (between 40 min and 24 hrs: $p \leq .001$; between 40 min and 48 hrs: $p \leq .001$; between 40 min and 72 hrs: $p \leq .001$) and for CMI 5 µM (between 40 min and 24 hrs, $p = .019$; between 40 min and 48 hrs, $p = .002$; between 40 min and 72 hrs, $p \leq .001$; between 48 hrs and 72 hrs, $p = .021$) **(B)**. *Analysis of azidobupramine effects on LC3-clustering; each data point represents the mean (±SEM) of three independent experiments averaging the signal of 10 distinct cells*: two-way ANOVA with repeated measures revealed main effects of group ($F(3, 8) = 54.21$, $p \leq .001$) and time ($F(3) = 17.38$, $p \leq .001$), and an interaction effect ($F(9) = 10.11$, $p \leq .001$); post-hoc group analyses (Bonferroni corrected) showed a higher clustering index for AZB 10 µM as compared to AZB 5 µM, AZB 1 µM, and DMSO after 24 hours (AZB 10 µM vs. AZB 5 µM: 24 hrs, $p \leq .001$; 48 hrs, $p = .003$; 72 hrs, $p = .006$; AZB 10 µM vs. AZB 1 µM: 24 hrs, $p \leq .001$; 48 hrs, $p \leq .001$; 72 hrs, $p = .002$; AZB 10 µM vs. DMSO: 24 hrs, $p \leq .001$; 48 hrs, $p \leq .001$; 72 hrs, $p = .001$); throughout the experiment, time-dependent differences were observed for AZB 10 µM (between 40 min and 24 hrs, $p \leq .001$; between 40 min and 48 hrs, $p \leq .001$; between 40 min and 72 hrs, $p \leq .001$) **(C)**. *Analysis of clomipramine effects on LC3-clustering; each data point represents the average of signal integration of 10 cells, each performed in triplicates*: Two-way ANOVA with repeated measures revealed main effects of group ($F(3, 8) = 192.07$, $p \leq .001$) and time ($F(3, 1.77) = 25.93$, $p \leq .001$), and an interaction effect ($F(9, 5.31) = 15.52$, $p \leq .001$); post-hoc group

analyses (Bonferroni corrected) showed higher clustering index for CMI10μM as compared to CMI5μM, CMI1μM, and DMSO after 24h (CMI10μM vs. CMI5μM: 24h, $p \leq .001$; 48h, $p = .001$; 72h, $p \leq .001$; CMI10μM vs. CMI1μM: 24, $p \leq .001$; 48h, $p \leq .001$; 72h, $p \leq .001$; CMI10μM vs. DMSO: 24h, $p \leq .001$; 48h, $p \leq .001$; 72h, $p \leq .001$). Moreover, CMI5μM had higher clustering index than CMI1μM at 24h ($p = .001$) as well as DMSO both at 40' ($p = .007$) and 24h ($p \leq .001$); throughout the protocol, time-dependent changes were observed for CMI10μM (between 40' and 24h, $p \leq .001$; between 40' and 48h, $p \leq .001$; between 40' and 72h, $p \leq .001$; between 24h and 48h, $p = .037$; between 24h and 72h, $p = .013$), CMI5μM (between 40' and 24h, $p \leq .001$; between 40' and 72h, $p = .022$) (**C**).
